# Cherenkov luminescence imaging is a fast and relevant preclinical tool to assess tumour hypoxia in vivo

**DOI:** 10.1186/s13550-018-0464-7

**Published:** 2018-12-20

**Authors:** Emiko Desvaux, Alan Courteau, Pierre-Simon Bellaye, Mélanie Guillemin, Camille Drouet, Paul Walker, Bertrand Collin, Richard A. Decréau

**Affiliations:** 1 Centre George François Leclerc (CGFL), 1 rue du Professeur Marion, 21079 Dijon, France; 2 Université Bourgogne Franche Comté, CNRS, Laboratoire Electronique Informatique & Image (Le2i), UMR, 6306 Dijon, France; 3 Université Hospital Francois Mitterrand, Dijon, France; 4 Institut de Chimie Moléculaire de l’Université de Bourgogne (ICMUB), 9 Avenue Alain Savary, 21078 Dijon, France

**Keywords:** Cherenkov luminescence imaging (CLI), ^18^F-Fluoromisonidazole (FMISO), Hypoxia, Positron emission tomography (PET), Magnetic resonance imaging, BOLD, Colon cancer

## Abstract

**Purpose:**

Molecular imaging techniques visualise biomarkers for both drug development and personalised medicine. In this field, Cherenkov luminescence imaging (CLI) seems to be very attractive by allowing imaging with clinical PET radiotracers with high-throughput capabilities. In this context, we developed a fast CLI method to detect tumour hypoxia with ^18^F-fluoromisonidazole (FMISO) for drug development purposes.

**Methods:**

Colon cancer model was induced in mice by subcutaneous injection of 1 × 10^6^ CT-26 cells. FMISO was injected, and simultaneous PET-blood oxygen level dependent (BOLD)-MRI followed by CLI were performed along with immunohistochemistry staining with pimonidazole.

**Results:**

There was a significant correlation between FMISO PET and CLI tumour uptakes, consistent with the BOLD-MRI mapping. Tumour-to-background ratio was significantly higher for CLI compared with PET and MRI. Immunohistochemistry confirmed tumour hypoxia. The imaging workflow with CLI was about eight times faster than the PET-MRI procedure.

**Conclusion:**

CLI is a fast and relevant tool to assess tumour hypoxia. This approach could be particularly interesting for hypoxia-targeting drug development.

**Electronic supplementary material:**

The online version of this article (10.1186/s13550-018-0464-7) contains supplementary material, which is available to authorized users.

## Introduction

Molecular imaging contributes to develop safer and effective drugs while shortening the time-to-market [1]. Cherenkov luminescence imaging (CLI) has emerged as a promising optical imaging (OI) modality. CLI is based on the intrinsic capability of radionuclides (e.g. fluorine 18) to emit light through the Cherenkov effect [2]. CLI shares the advantages of OI with high sensitivity, low cost and high throughput [2]. CLI is widely used in preclinical oncology, although it has never been used to investigate tumour hypoxia. Hypoxia is encountered in aggressive tumours and is responsible for the resistance to treatments [3]. The tumour hypoxic status can be determined with ^18^F-fluoromisonidazole (FMISO) positron emission tomography (PET) and/or blood oxygen level dependent (BOLD) magnetic resonance imaging (MRI) [3]. Albeit appealing in several regards, such techniques suffer from limited throughputs [4]. Indeed, PET/MRI imaging requires expensive systems and is usually characterised by measurement times ranging from 20 to 30 min for static imaging studies and up to 60–90 min when involving dynamic PET assessments or/and functional MRI imaging [4]. Thus, CLI could represent a fast, cost-effective and decisive tool to predict the efficacy of hypoxia-activated anticancer drugs since baseline hypoxia imaging is of crucial interest to successfully develop hypoxia-targeted drugs in both preclinical [5] and clinical settings [6]. In addition, the use of CLI in humans has also recently been demonstrated, providing CLI with an important translational aspect [7]. Herein, we present the first report of preclinical hypoxia imaging by CLI and its cross-validation by simultaneous PET-MRI imaging following injection of FMISO.

## Materials and methods

### Animal experiments

Animal experiments were approved by our institution (Centre Georges-François Leclerc, Dijon, France) and complied with the Ethical Committee and the French Ministry of Higher Education and Research. Six- to 7-week-old-BALB/c female mice (*n* = 13) were obtained from the animal husbandry of the University of Burgundy (Dijon, France). A total of 1 × 10^6^ murine CT26 (ATCC, CRL-2638, USA) colon cancer cells were implanted subcutaneously at the right flank of depilated mice. The tumour grew over 18 days until it reached a mean volume of 316 ± 81 mm^3^. Then, mice were injected intravenously under anaesthesia (2% isoflurane in air) with 10 MBq of FMISO and pimonidazole (60 mg/kg). PET-MRI imaging was performed 120 min post-injection (p.i.), and CLI imaging was performed on the same animal right after the PET-MRI exam (160 min p.i.). The tumour area was depilated 24 h before CLI imaging. All images were decay corrected for quantification. After the imaging sessions, mice were sacrificed and tumours were formalin-fixed (48 h) for pimonidazole immunohistochemistry. ^18^F-Fluoromisonidazole (FMISO) was synthesised by Pharmimage® within its cyclotron and radiopharmacy platform following an in-house synthetic scheme (Additional file 1).

### PET-MRI imaging

PET and MRI images were simultaneously acquired in a dual-ring SiPM microPET fully integrated in a 7 T preclinical MRI (MR Solutions, Guildford, UK). During imaging, mice were anaesthetised with 2% isoflurane in air. In addition to T1 anatomical images, BOLD-MRI sequences have been performed using multi-gradient echo (MGE) sequence (T2*-weighted acquisition) with the following parameters: TR = 500 ms; TE = 3, 6, 9, 12, 15 and 18 ms; flip angle = 40°; and image matrix = 128 × 128. MRI voxel values have been interpolated using a Python 3 homemade script, to obtain a T2* map (BOLD data). PET images have been reconstructed using a 3D OSEM algorithm (2 iterations, 32 subsets). PET quantifications were performed using Vivoquant™ software (Invicro, Boston, MA, USA), and results have been expressed in % injected activity of FMISO per mm^3^ (%IA/mm^3^).

### CLI imaging

Following PET-MRI imaging, mice underwent CLI imaging performed with an optical imager (IVIS Lumina III®, PerkinElmer, USA) equipped with a CCD camera with the following parameters: exposure time 300 s, binning factor 16, field of view 7.5 cm, in open filter mode. Mice were maintained under anaesthesia throughout the procedure (2% isoflurane in air). Results are expressed in radiance (p/s/cm^2^/sr). Signal-to-noise ratio for PET, MRI and CLI following the injection of FMISO was determined by the ratio of the signal from the tumour and a contralateral irrelevant region of interest (muscle).

### Pimonidazole immunostaining

Tumour sections from mice injected with pimonidazole (given at 60 mg/kg i.p., 120 min before sacrifice) were deparaffinised and immunostained with a commercial kit (Hydoxyprobe plus kit, USA) to specifically stain pimonidazole and determine the level of hypoxia. Stained sections were analysed using Axioscope A1® coupled to an Axiocam 503® colour camera and ZEN® Software (Zeiss, Germany).

### Statistical analysis

For all the experiments performed within this study, statistical analyses were achieved using GraphPad Prism® 7.0 (GraphPad Software, USA) and a *p* value of less than 0.05 was considered significant. Comparisons between more than two groups have been performed using one-way ANOVA, and correlations were determined by the Pearson’s coefficient using GraphPad Prism® 7.0.

## Results

### CLI imaging of tumour hypoxia and cross-validation with simultaneous PET-MRI

**Fig. 1:**
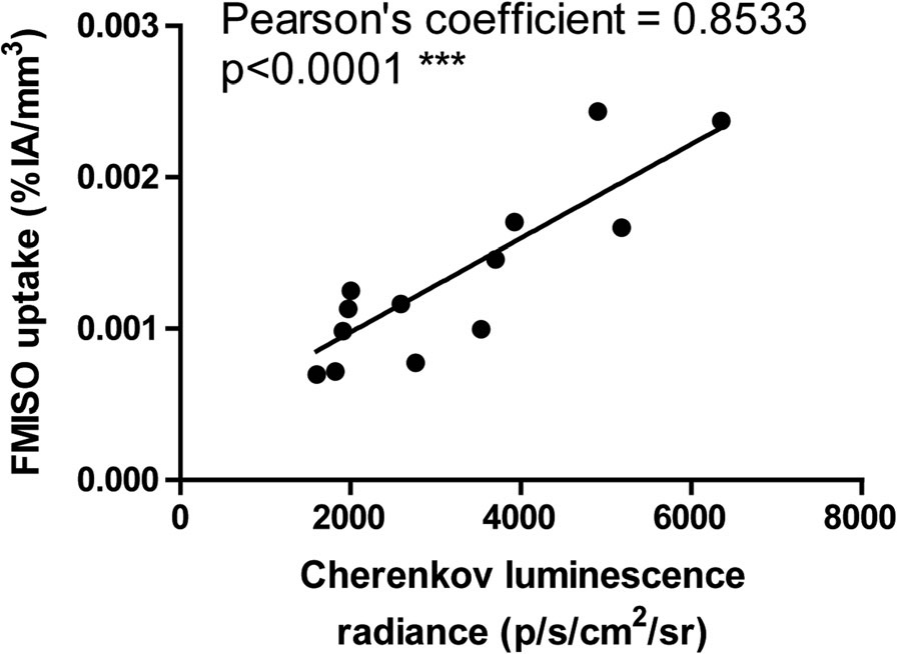
Correlation between Cherenkov luminescence imaging and PET following the injection of FMISO (*n* = 13, Pearson’s coefficient = 0.8533, *p* < 0.0001***). Results are presented as radiance (p/s/cm^2^/sr) for CLI and %IA/mm^3^ for FMISO-PET

**Fig. 2:**
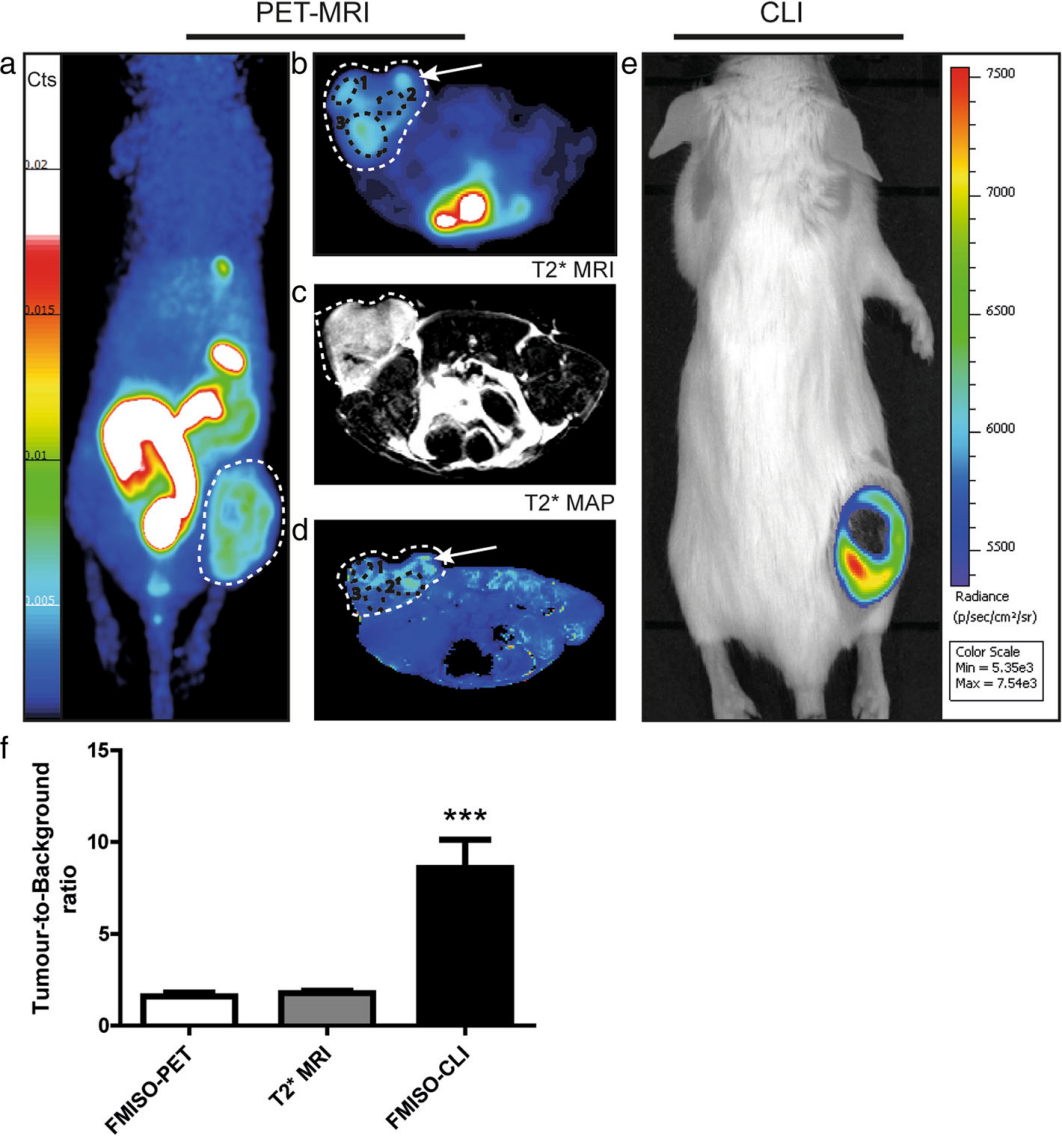
Imaging of hypoxia within CT26 tumour bearing mice (*n* = 13), PET-MRI **a**–**d** Representative PET-MRI images showing the global co-localisation of FMISO uptake and BOLD MRI signal. Images were acquired 120 min post-injection of 10 MBq of [^18^F]-Fluoromisonidazole (FMISO). PET. **a** Representative maximum-intensity-projection FMISO PET Image; **b** Transversal slice showing FMISO uptake within the tumour; **c** T2*-weighted MRI and **d** BOLD image derived from T2* mapping. White dashed lines: tumour limits, white arrows: oxygenated tumour area (increased BOLD signal), black circle: hypoxic tumour areas (decreased BOLD signal). CLI (**e**): Representative FMISO CLI image of the same mouse as for **a** to **d** acquired just after the PET-MRI scan. **f** Tumour-to-background ratio for PET, MRI and CLI following the injection of FMISO determined by the ratio of the signal from the tumour and a contralateral irrelevant region of interest (muscle); *n* = 13, ***p < 0.001

The tumour uptake of FMISO was measured with both PET and CLI. Through our work, we managed to highlight a positive correlation (*n* = 13; Pearson’s coefficient = 0.8533, *p* < 0.0001) between FMISO uptake obtained with PET and CLI (Fig. 1). No matter what imaging modality was used, all tumours exhibited hypoxia in our model as shown by both FMISO tumour uptake measured by PET (Fig. 2a, b) and BOLD MRI mapping (Fig. 2c, d) or CLI (Fig. 2e). The BOLD signal obtained from T2*-weighted images reflects the heterogeneity in the magnetic field B_0_ caused by changes in the level of O_2_ in the blood. The signal decrease on T2* maps hence reflects deoxyhaemoglobin paramagnetic characteristics, thus providing indirect information about haemoglobin saturation rate, reflective of tumour oxygenation. Interestingly, we demonstrated that hypoxic areas within tumours measured with FMISO-PET corresponded with the area of decreased BOLD signal obtained with the MRI (Fig. 2b, d, black circle). Nevertheless, some FMISO-PET positive areas showed corresponding increased BOLD signal reflecting properly oxygenated areas thus highlighting discrepancies between cellular hypoxia measured by FMISO-PET and blood oxygen levels measured by MRI (Fig. 2b, d, white arrow). Most importantly, while the overall PET-MRI procedure took approximately 40 min per animal (39.85 ± 1.81 min), the CLI procedure only lasted for 5 min per animal. Tumour-to-background ratio (TBR) has been measured for all imaging modalities, and most interestingly, CLI displayed a significantly higher TBR demonstrating the sensitivity of this imaging technique (Fig. 2f).

### Hypoxia immunostaining with pimonidazole

**Fig. 3:**
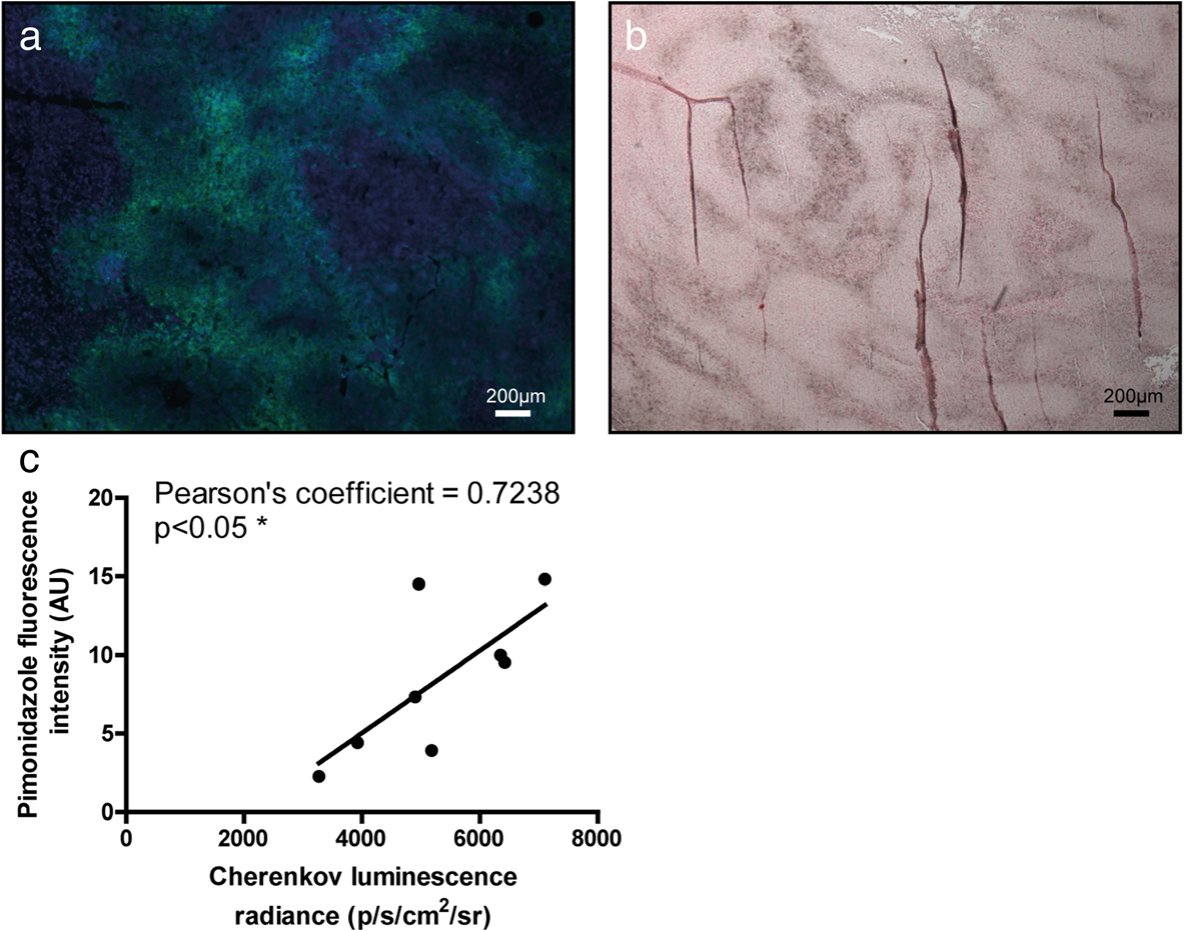
Microscopic slides of tumours for the same mouse as for Fig. 2a, b with pimonidazole staining (× 50 magnification). Hypoxic regions are shown either in green (**a** fluorescence microscopy) or in brown (**b** transmitted light microscopy). **c** Correlation between Cherenkov luminescence imaging following the injection of FMISO and fluorescence pimonidazole staining (*n* = 9, Pearson’s coefficient = 0.7238, *p* < 0.05*). Results are presented as radiance (p/s/cm^2^/sr) for CLI and fluorescence intensity (arbitrary units, AU) of pimonidazole

The ex vivo study performed on slices of CT26 colon carcinoma further confirmed CLI-PET-MRI findings showing diffused hypoxic areas heterogeneously distributed within the tissue (Fig. 3) with either fluorescence microscopy (Fig. 3a) or transmitted light microscopy (Fig. 3b). Interestingly, we demonstrated a moderate correlation (*n* = 9; Pearson’s coefficient = 0.7238, *p* < 0.05) between pimonidazole fluorescence microscopy and FMISO-CLI (Fig. 3c).

## Discussion

Biomarkers of tumour hypoxia allow to improve the drug development process through the selection of individuals potentially eligible for therapies that target hypoxic environment in both preclinical and clinical settings [5]. FMISO is a well-known PET radiotracer of tumour hypoxia [3] able to produce a CR due to the emission of a positron with a maximal energy of 633 keV [2]. The present study is the first report of CLI showing the FMISO uptake in hypoxic tumours, cross-validated by simultaneous PET-T2* MRI, further confirmed by classical pimonidazole immunostaining [3]. We assume that the advantages of CLI are twofold: (i) fast imaging since the acquisition time for simultaneous PET-MRI imaging was about 40 min, whereas that of CLI was as low as 5 min and (ii) CLI signal remains proportional to the PET signal since both significantly correlated, consistent with previous data [2, 8]. Our results are in accordance with other studies, which demonstrated a clear linear correlation between CLI radiance and PET using several radionuclides including 18-fluorine, 64-copper or 89-zirconium in vivo [9–11]. A close correlation between CLI and γ-counter-based biodistribution analysis has also been highlighted by several studies further validating this fast and cheap technique as a reliable alternative for high-throughput analysis [9, 11]. However, CLI imaging suffers from limitations. Compared with PET/MRI, which provides a 3D image, CLI only gives a 2D image hampering accurate quantification of the signal. In addition, CLI-based imaging relies on limited tissue penetration and low sensitivity for deep targets as well as the high amount of diffusely scattered photons in the mouse tissues. Therefore, FMISO-CLI only gives a qualitative measure while FMISO-PET gives a fully 3D quantitative measurement of tumour hypoxia. Nevertheless, our study demonstrates that CLI provides favourable tumour-to-background ratio for shallow signals (e.g. subcutaneous tumours), compared with that of PET and MRI. As a consequence, FMISO-CLI appears as a sensitive and powerful tool to rapidly and cost-effectively discriminate subcutaneous tumours with hypoxia from tumours without hypoxia in the context of hypoxia-targeting drug discovery. Importantly, the improved workflow brought by CLI not only provides faster preclinical screening of hypoxia, but also considerably reduces the time each animal remains under anaesthesia, which is a critical parameter when evaluating tumour hypoxic status. As a consequence, CLI may provide more reliable data regarding baseline hypoxia, which in turn may improve hypoxia-related drug development. Given the relative heterogeneity of hypoxic signals among animals/patients in tumours in both clinical and preclinical settings, it appears crucial that the development of hypoxia-targeting drugs relies on fast and relevant biomarkers of hypoxia. Such a strategy involving FMISO-CLI would allow a rapid selection of subjects with positive hypoxic signal in order to successfully evaluate hypoxia-targeting drugs. This was recently highlighted by Grkovski et al. who suggested that the poor outcome of the MAESTRO trial, evaluating the hypoxia-activated evofosfamide, was partly due to the inclusion of non-hypoxic tumours, which may have masked the benefit of the drug [5]. Such a result shows that not only CLI is relevant to assess tumour hypoxia, but also for preclinical drug development due to its high throughput capabilities also reported elsewhere [8]. Strategies are underway to make CLI an even more potent technique for oncologic studies [12].

An innovative aspect of our study relies on the application of CLI to tumour hypoxia and the cross-validation with classical pimonidazole immunostaining. As mentioned previously, correlation between CLI and PET is already documented but, to our knowledge, the current study is the first to validate CLI as an accurate tool to quickly detect tumour hypoxia. The correlation between CLI and pimonidazole immunostaining that we demonstrate, while significant, is weaker than that between CLI and PET. The major issue with classical immunostaining quantification, widely used in clinic, relies on the fact that it is only representative of a small portion of the tissue potentially introducing bias in analysis. Tumour hypoxia is heterogeneously distributed within tumours and is often subjected to rapid changes depending on various environmental parameters (e.g. temperature, activity status of the animal). Therefore, rapid and non-invasive evaluation of tumour hypoxia is crucial to obtain the most accurate signal. Histological measurement of hypoxia requires to euthanize animals, resect the tumour and then fix the tissue in formalin-based solution. Even though these procedures may only require a short period of time, the hypoxic status of the tumour may suffer from some changes (e.g. short lack of oxygen following animal euthanasia). Therefore, we believe that CLI imaging of FMISO is a more representative measure of in vivo tumour hypoxia compared with histology.

Important questions can be raised about the clinical applicability of CLI in humans given the differences between preclinical and clinical studies regarding, for instance, injected doses and depth of the sites of interest. In our preclinical study, CLI imaging was performed on a subcutaneous tumour after injection of 10 MBq of FMISO (400 MBq/kg in a mouse of 25 g). As a comparison, current guidelines prescribe a dose of 3.7 MBq/kg in patients for standard PET exams. With a 100-fold lower injected activity and deeper tumour site, CLI imaging in humans remains a challenge and often requires the use of ultrasensitive photon-detecting devices and strict restriction of ambient light [7]. Nevertheless, the proof-of-concept for the clinical use of CLI has already been described especially in the field of guided surgery [13] where tumour hypoxia might be a relevant target [14]. The use of CLI in the context of guided surgery may indeed represent a way to circumvent the limited tissue penetration and tissue diffusion of CR. Based on our results, FMISO-CLI could therefore be a fast, cheap and sensitive method to specifically and accurately remove otherwise undetectable hypoxic malignant lesions. In addition, with the recent rise of intraoperative radiation therapy, the detection of radio-resistant hypoxic tumour areas by imaging such as FMISO-CLI might be of great interest in order to provide radiation boosts which would improve radiation therapy outcome [15].

## Conclusion

Tumour hypoxia can be assessed with CLI induced by FMISO since it is cross-validated by PET-MRI and histology. Interestingly, the workflow for CLI imaging is much more efficient than PET-MRI paving the way for this method to speed-up and strengthen the preclinical development of hypoxia-targeting drugs.

## Additional file

Additional file 1: Synthesis and purification of ^18^F-Fluoromisonidazole (FMISO). (DOCX 344 kb)
