# Supplementary material for: Cherenkov luminescence imaging is a fast and relevant preclinical tool to assess tumour hypoxia in vivo
Source: EJNMMI Res. 2018 Dec 20;8:111. doi: 10.1186/s13550-018-0464-7 (PMC6301908; doi:10.1186/s13550-018-0464-7)
Supplement: Supplementary file 1 — Synthesis and purification of 18F-Fluoromisonidazole (FMISO). (DOCX 344 kb) [file 13550_2018_464_MOESM1_ESM.docx]

Experimental setup

1. Reagents and apparatus

NITTP was obtained from ABX Advanced Biomedical Compounds. The 4,7,13,16,21,24-hexaoxa-1,10-diazabicyclo[8.8.8]hexacosane [Kryptofix 222 (K222)] was obtained from Sigma-Aldrich. QMA and Alumina Sep-Pak cartridges were obtained from Waters (Milford, MA, USA). All reagents were used without further purification. The Tracerlab FX2N synthesis module was purchased from GE Healthcare. HPLC for [^18^F]FMISO purification was carried out in the Tracerlab FX2N synthesis module built-in HPLC system with a semipreparative reversed-phase C-18 column and C-18 precolumn equipped with a UV detector and a radioactivity detector. For quality control, HPLC analysis was carried out on a modular HPLC system with a reversed phase analytic C-18 column (150x4.6 mm).

1. [^18F^]FMISO synthesis module

[^18^F]FMISO was synthesized in a commercially available Tracerlab FX2N synthesis module. The module was operated and monitored via a process control box connected to a computer. The synthesizer was programmed by the software package in a step-by-step time-dependent sequence of events such as valve opening or closing, helium purging, vacuum, temperature going up/down and HPLC flow rate increase/decrease. Several live parameter records were performed during the synthesis process, including activity in the target vial, reaction vessel and product vial; temperature and pressure in the reactor; and UV absorbance and radioactivity measurements for the semipreparative HPLC.


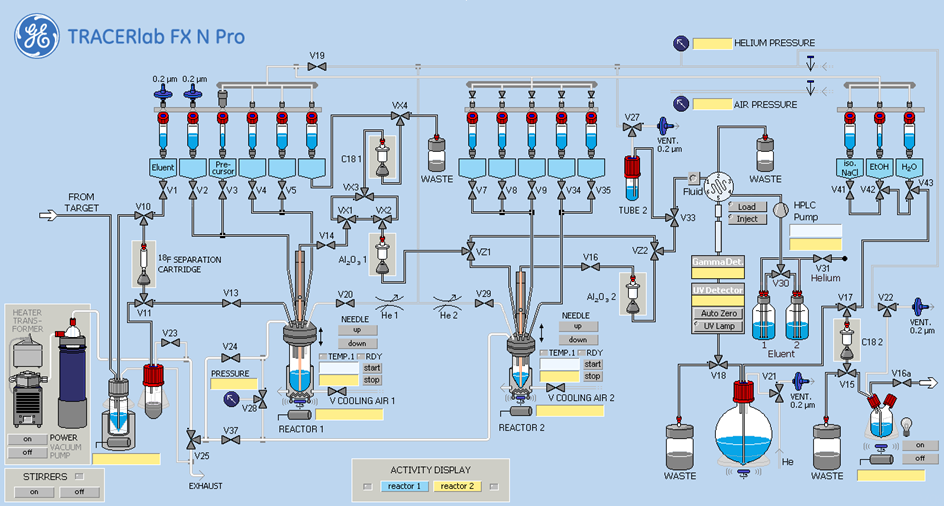


1. Automated synthesis of [^18^F]FMISO

The automated radiochemical synthesis of [^18^F]FMISO was a two-step, onepot procedure that consisted of ^18^F-fluorination of NITTP and subsequent hydrolysis of the THP-protected product on 1-(2V-nitro-1V-imidazolyl)-2-O-tetrahydropyranylpropanol as the precursor.


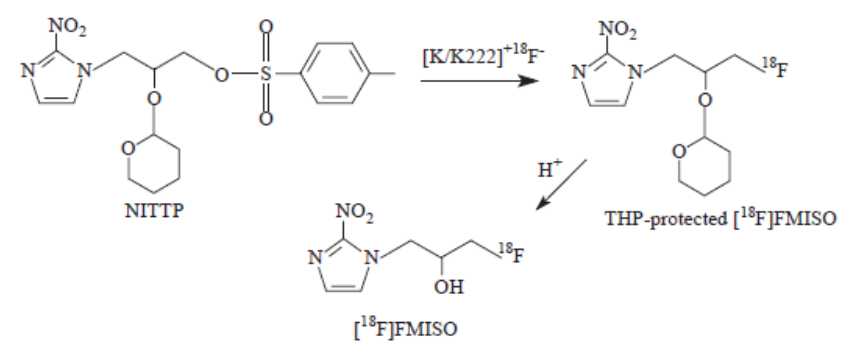


Steps for the preparation of [^18^F]FMISO consisted of three categories as listed in Table 1. The individual synthesis steps involved in the preparation of [^18^F]FMISO can be divided into three main categories: fluorination of the precursor, hydrolysis of the protective group and neutralization/HPLC purification. After synthesis, cleaning procedures were carried out in between the runs.

Before delivery of [^18^F]fluoride to the module, Vial 1 was filled with a mixture of 15 mg of K222, 3 mg of K_2_CO_3_, 1 ml of acetonitrile and 0.5 ml of water; Vial 3 was added with 5 mg of the precursor NITTP dissolved in 1 ml of DMSO; Vial 4 was added with 1 ml of 1N HCl; Vial 5 was filled with 0.5 ml of 30% sodium acetate; and Vial 6 was added with 1 ml of HPLC eluent.

[^18^F]fluoride was obtained through the nuclear reaction ^18^O(p, n)^18^F by irradiation of a 95% ^18^O-enriched water target with a proton beam at a PETtrace cyclotron. After the delivery of [^18^F]fluoride from the cyclotron, the radioactivity was collected on a QMA Sep-Pak cartridge where [^18^F]fluoride was trapped and 1^8^Owater was collected for recycling. A total of 1.5 ml of a K222 solution in Vial 1 was eluted through the QMA Sep-Pak cartridge, in which the trapped ^18^F^-^ was eluted into the reaction vessel. The solvent was evaporated under a stream of nitrogen. After complete removal of the solvent, the precursor in Vial 3 was added to the reaction vessel containing the dried [K/K222]+^18^F^-^ complex, and the vessel was heated for 10 min at 100°C. Then, from the resulting reaction mixture was added to 1N HCl in Vial 4. The mixture was hydrolyzed to remove the THP-protected group by heating for 5 min at 100°C. For HPLC purification, the mixture was neutralized with 30% sodium acetate and passed through an alumina Sep-Pak cartridge. The eluate was collected in a glass vial. Before HPLC purification, 1 ml of HPLC eluent (H_2_O/C_2_H_5_OH 95/5, vol/vol) in Vial 6 was added to the reaction vessel and the solution was then passed through the same alumina Sep-Pak cartridge. The eluate was collected in the same glass vial. Finally, [^18^F]FMISO was purified by HPLC system consisting of a pump, an automatic sample injector, a reversed phase C-18 column (250x16 mm), a UV absorption detector and a NaI-radiodetector. The mobile phase used was H_2_O/C_2_H_5_OH (95/5, vol/vol) at a flow rate of 8 ml/min. The peak corresponding to [^18^F]FMISO was collected. 10% NaCl was added to obtain the final formulation.

Table 1

Summary of operation steps in the module category step

**Fluorination of the precursor**

A. [^18^F]fluoride trapped by QMA cartridge

B. Addition of eluent to elute [^18^F]fluoride from QMA cartridge

C. Drying of [^18^F]fluoride

D. Addition of precursor solution to reaction vessel

E. [^18^F]fluorination at 100°C for 10 min

**Hydrolysis of protective groups**

F. Addition of 1N HCl for hydrolysis

G. Hydrolysis at 100°C for 5 min

**HPLC purification and formulation**

H. Neutralization with 30% sodium acetate

I. Addition of HPLC eluent to reaction vessel

J. Transfer of the solution to HPLC loop

K. Elution using EtOH/H2O=5:95; flow, 8 ml/min; detection, UV 313 nm

L. Collection of the radioactive peak of [18F]FMISO (retention time, 15–19 min)

N. Formulation with 10% NaCl

1. Quality control and stability

The analytic HPLC was used for checking of radiochemical purity and specific activity eluted with H_2_O/CH_3_CN (93/7, vol/vol) at a flow rate of 1 ml/min.
